# Association between metabolic syndrome severity score and cardiovascular disease: results from a longitudinal cohort study on Chinese adults

**DOI:** 10.3389/fendo.2024.1341546

**Published:** 2024-04-08

**Authors:** Jing-jing Lin, Pin-yuan Dai, Jie Zhang, Yun-qi Guan, Wei-wei Gong, Min Yu, Le Fang, Ru-ying Hu, Qing-fang He, Na Li, Li-xin Wang, Ming-bin Liang, Jie-ming Zhong

**Affiliations:** Department of Chronic and Non-communicable Disease Prevention and Control, Zhejiang Provincial Center for Disease Control and Prevention, Hangzhou, China

**Keywords:** metabolic syndrome, cardiovascular disease, heart disease, stroke, cohort study, hazard ratio

## Abstract

**Objective:**

This study aimed to quantify the severity of metabolic syndrome(MetS) and investigate its association with cardiovascular disease(CVD) risk on Chinese adults.

**Methods:**

13,500 participants from the Zhejiang Adult Chronic Disease Study were followed up between 2010 and 2021. A continuous MetS severity score derived from the five components of MetS was used to quantify MetS severity, and the association between MetS severity and the risk of incident CVD was assessed using Cox proportional hazard and restricted cubic spline regression.

**Results:**

Both the presence and severity of MetS were strongly associated with CVD risk. MetS was related to an increased risk of CVD (hazard ratio(HR):1.700, 95% confidence interval(CI): 1.380–2.094). Compared with the hazard ratio for CVD in the lowest quartile of the MetS severity score, that in the second, third, and highest quartiles were 1.812 (1.329–2.470), 1.746 (1.265–2.410), and 2.817 (2.015–3.938), respectively. A linear and positive dose-response relationship was observed between the MetS severity and CVD risk (*P* for non-linearity = 0.437). Similar results were found in various sensitivity analyses.

**Conclusion:**

The MetS severity score was significantly associated with CVD risk. Assessing MetS severity and further ensuring intervention measures according to the different severities of MetS may be more useful in preventing CVD.

## 1 Introduction

Metabolic syndrome (MetS) comprises a set of metabolic disturbances, including abdominal obesity, elevated blood pressure, glucose intolerance, insulin resistance, and dyslipidemia (1, 2). MetS is highly prevalent worldwide and has become a crucial public health issue because of its detrimental effects on human health (3–5). A national survey in China revealed that 31.1% of residents aged ≥20 years experienced MetS, and the prevalence of MetS is still increasing, especially among women, people aged ≥45 years, and urban residents (6), inducing a substantial burden on public health.

MetS has been related to numerous adverse health outcomes, including diabetes, chronic kidney disease, and colorectal cancer (7–9). MetS can increase the risk of a series of cardiovascular outcomes, such as stroke, coronary heart disease, and heart failure (10, 11). A meta-analysis including 87 prospective observational studies showed that MetS is associated with a 2.35- and 2.40-fold increased risk of CVD events and mortality, respectively (12). Considering the significant negative impact of CVD on human health and lifespan, early identification and assessment of MetS is crucial.

Notably, several widely used diagnostic criteria for MetS have been proposed by institutions such as the National Cholesterol Education Program Adult Treatment Program III (ATP-III), the International Diabetes Federation and the World Health Organization, which classify patients as having or not having MetS based on the number of component abnormalities (13). However, the clinical criteria and levels of each component for identifying MetS vary according to countries or institutions; therefore, unified diagnostic criteria for MetS are currently lacking. Furthermore, some data may be lost in the binary classification of MetS, resulting in an undetermined underestimation of risk (13, 14). Consequently, continuous scoring systems for MetS have been increasingly proposed over the past few years to measure MetS severity and dynamically follow up on individuals for the degree of change in MetS over time (15–17).

To date, most previous studies on the relationship between MetS and CVD risk have treated the presence of MetS as a dichotomous variable, whereas only a few studies have used MetS severity scores to explore the association between MetS severity and CVD events (18–21). Several studies had indicated that continuous score of MetS can provide valuable information in predicting coronary heart disease, myocardial infarction and stroke and have greater potential for predicting cardiovascular disease than traditional criteria of MetS (19, 20, 22). In addition, a continuous system of MetS enables dynamic assessment of changes in disease and risk of CVD, which can further provide guidance for CVD prevention. As far as we know, evidence regarding the contribution of MetS severity to CVD risk on Chinese population remains insufficient. Therefore, this study aimed to examine the association between the MetS severity score and incident CVD in Chinese adults based on an 11-year follow-up longitudinal study.

## 2 Materials and methods

### 2.1 Study design and population

This cohort study used a subset of the Zhejiang Adults Chronic Disease Study, an ongoing cohort study in Zhejiang, China. In this study, 19,113 participants aged ≥18 years in 7,571 households were recruited from 15 counties and 180 villages or communities in Zhejiang province between July 2010 and November 2010, using the multistage stratified cluster sampling method. Exclusion criteria were age <18 years, dementia or schizophrenia, bedridden illness, deafness or dumbness, <6 months living in the local area, and inability or unwillingness to sign a consent form (23). This study was approved by the Ethics Committee of Zhejiang Provincial Center for Disease Control and Prevention. And all participants gave informed consent to participate before taking part.A total of 17,437 participants completed standard questionnaires on demographic information, lifestyle, and health status and the baseline survey response rate was 91.23%. Physical examinations and laboratory tests were also conducted to assess height, weight, waist circumference (WC), systolic blood pressure (SBP), diastolic blood pressure (DBP), triglycerides (TC), fasting blood glucose (FBG), and high-density lipoprotein cholesterol (HDL-C). WC and BP were measured at least twice by trained interviewers, and the values were averaged. Mean arterial pressure (MAP) was calculated by the formula: MAP = 1/3SBP + 2/3DBP. CVD events or death outcomes were monitored annually. This study adhered to the Strengthening the Reporting of Observational Studies in Epidemiology (STROBE) guidelines (24).

**Figure 1:**
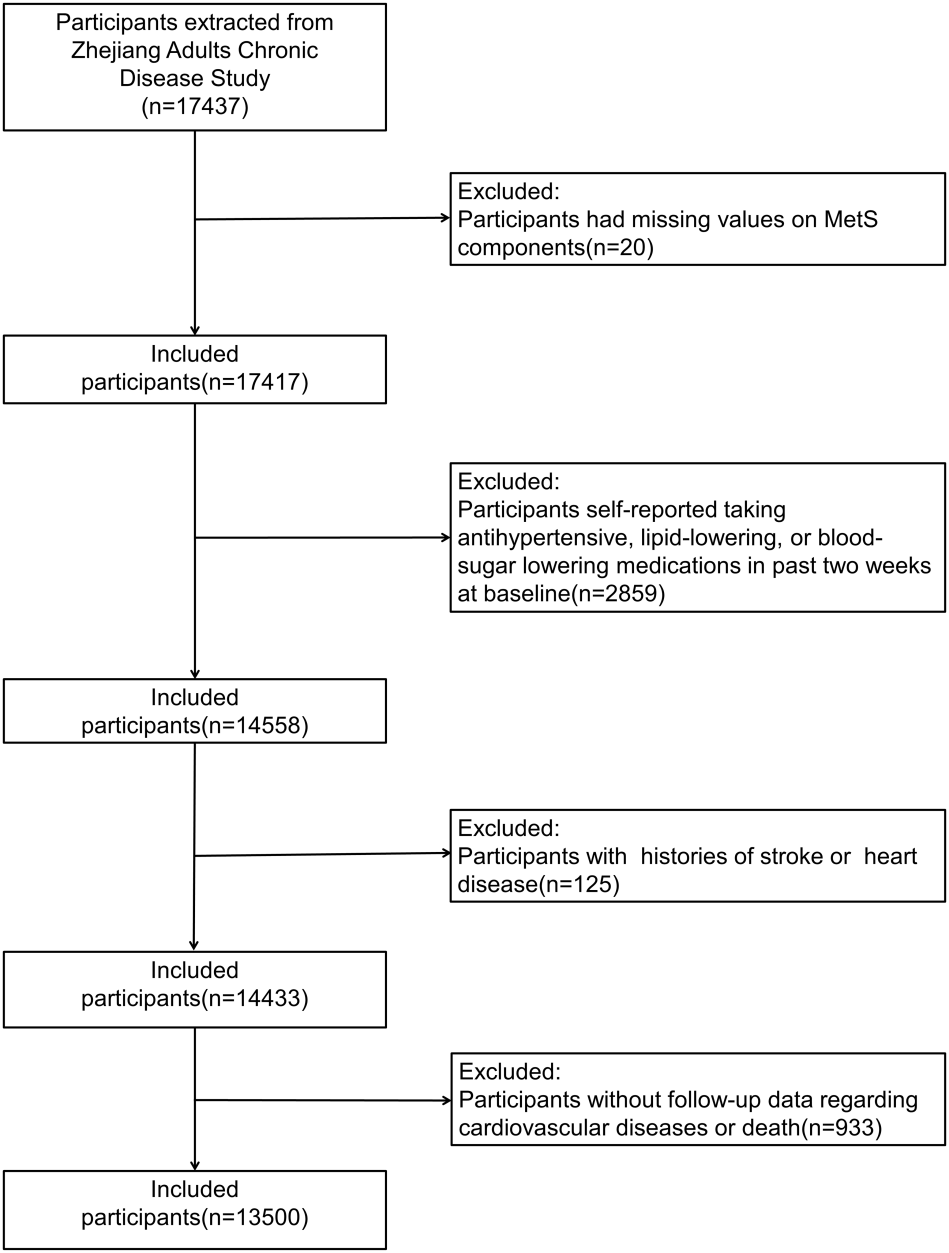
Study profile.

Participants with missing values on MetS components (n = 20), those who self-reported taking antihypertensive, lipid-lowering, or blood-sugar-lowering medications in the past 2 weeks at baseline (n = 2859), those with a history of stroke or heart disease (n = 125), and those without follow-up data on CVD or death (n = 933) were further excluded from this study. Finally, 13,500 participants were included in this study (**Figure 1**).

### 2.2 Definition and assessment of MetS severity

In this study, the ATP-III criteria modified by the American Diabetes Association (ADA) were used to diagnose MetS, which had been described elsewhere (1). To assess MetS severity, we used the MetS components from the ATP-III criteria to establish a continuous MetS severity score system and calculated individual MetS severity scores, as previously described (15). Briefly, we first normalized five components: WC, BP (represented by MAP), TC, HDL-C, and FBG. Then, principal component (PC) analysis (varimax rotation) was performed on these components to derive PCs with eigenvalue ≥1.0, representing large fractions of MetS score variance. Finally, the MetS severity score was computed by summing the individual scores of all PCs weighted by the relative contribution of each PC in the explained variance. We derived two PCs, accounting for 30% and 29% of the variance, respectively (loadings PC1 [PC2]: WC 0.57 [0.53], TG 0.34 [0.68], HDL 0.21 [-0.85], MAP 0.76 [0.07], and FBG 0.67 [−0.01]).

### 2.3 Study outcomes

We collected data on all acute CVD and death events that occurred during the follow-up period from the Zhejiang Provincial CVD Surveillance System and matched the outcomes based on information including participants’ names, sex, identity numbers, and addresses. The surveillance system monitored data on all-cause deaths and incident CVD events reported by all levels of hospitals in Zhejiang Province. Besides, death certificate and supplementary investigation are complementary methods of collecting data on acute CVD events (25). According to the 10^th^ revision of the International Classification of Diseases (ICD-10), the subtypes of CVD in the present study were stroke (ICD-10 codes: I60–I63) and heart disease (ICD-10 codes: I21–I25). CVD was diagnosed by at least two doctors and reviewed by the Centers for Disease Prevention and Control (CDC) at the county level. The provincial CDC was responsible for quality control and regularly verifying the accuracy of the data. The study population was followed up until the date of CVD events, death, or December 31, 2021, whichever was first. The median follow-up period in this study was 11.3 years.

### 2.4 Covariates

A standard questionnaire was administered to obtain participants’ information about health-related factors and sociodemographic status, including age, sex, residence, marriage, education level, marital status, and annual income. Education level was divided into four levels: no formal education, primary school, secondary school, and college or above. Marital status was classified as never married, married, divorced, or widowed. Health-related factors included a history of heart disease and stroke, a history of using anti-hypertension, antidiabetics, or lipid-lowering drugs, and smoking and drinking status. Data on the history of heart disease or stroke was obtained by asking the following question: “Have you ever been diagnosed with a heart disease or stroke?” Smoking or drinking status was classified as current smoker/drinker, former smoker/drinker, or never smoker/drinker. We calculated body mass index (BMI) by dividing weight by the square of height.

### 2.5 Statistical analysis

The statistical analysis was performed for the period between March 10, 2023 and June 30, 2023. Mean ± standard deviation and median (inter-quartile range) were used to describe the distribution of normally distributed, and non-normally distributed data respectively. Categorical variables were presented as frequencies (percentages). The MetS score was divided into four quartiles and groups. Baseline characteristics were summarized according to different levels of MetS severity scores and further compared by the chi-square test, analysis of variance or the Kruskal–Wallis test, respectively for categorical variables, normally distributed continuous variables and non-normally distributed continuous variables. Notably, 4.65% (628 of 13,500) of all participants had missing values on at least one item, which were assumed to be missing at random. Therefore, multiple imputation method was employed to fill in missing data on age, sex, income, education level, marital status, smoking and drinking status. We created 5 imputed data sets by the predictive mean matching method and pooled the results using “mice” package in R version 4.3.0.

The follow-up period was calculated from the date of the baseline survey (July–November 2010) to the date of CVD events, death, or the end of follow-up (December 31, 2021), whichever was first. We computed the incidence density of CVD per 1000 person-years according to different levels of MetS score, and used Cox proportional hazards models to assess the association between MetS severity score and CVD risk. The assumption of proportional hazards was examined by Schoenfeld residual test, and no significant deviation from the assumption was found in all Cox analysis. The importance of each component in the full model was estimated using the partial chi-square statistic minus the predictor degrees of freedom(*x* ^2^-df). We also adopted the ATP-III criteria modified by the ADA to ascertain MetS and assessed the association between the presence of MetS and CVD risk. In addition, four-knotted restricted cubic spline regression was used to explore the nonlinear associations. Furthermore, subgroup analyses were conducted to test for effect modification by age(<50 or ≥50 years old), sex, residence, BMI(≤23.9 or ≥23.9), smoking and drinking status. Interaction on the multiplicative scale was evaluated using likelihood ratio tests.

Three sensitivity analyses were performed as follows: (1) using the complete dataset without missing data(12,855 participants) and repeating all analyses. A comparison of basic characteristics between the included and excluded groups was presented in **Supplementary Table S1** in **Supplementary Material**. (2) using the sample excluding participants who experienced CVD within the first 2 years of follow-ups and repeating all analyses; and (3) using the Fine-Gray competing risk models to measure competing risks of mortality (26). R version 4.3.0 (http://www.R-project.org) was used for all analyses. Statistical significance was set at *P* < 0.05.

## 3 Results

### 3.1 Baseline characteristics of the study population

**Table 1** presents participants’ baseline characteristics according to the MetS severity score. The median (inter-quartile range) age of the 13,500 participants at baseline was 47 (21) years. Of the participants, 47.46% were men, and 36.90% lived in the city. The univariate analysis revealed that participants with higher MetS scores tend to be older, be males, live in the city, have a lower education level, have a marital history, have a higher income, be current or former smokers, be current or former drinkers, and have a higher BMI.

**Table 1:** Baseline characteristics of 13500 participants according to metabolic syndrome score.

Characteristics	Participants, No.(%)					*χ^2^/H*	*P* value
	Total Sample (N=13500)	Metabolic syndrome score					
		Q1(<-0.432)	Q2(-0.432~0.037)	Q3(0.038~0.383)	Q4(≥0.384)		
Age, median(inter-quartile range)	47(21)	41(25)	47(20)	49(19)	50(17)	572.027	<0.001
Men	6407(47.46)	1264(19.73)	1584(24.72)	1636(25.53)	1923(30.01)	258.734	<0.001
City residence	4982(36.90)	1184(23.77)	1245(24.99)	1257(25.23)	1296(26.01)	8.063	0.045
Education level							
No formal education	2257(16.72)	427(18.92)	590(26.14)	614(27.20)	626(27.74)	247.789	<0.001
Primary school	4290(31.78)	900(20.98)	1096(25.55)	1158(26.99)	1136(26.48)		
Secondary school	6255(46.33)	1749(27.96)	1515(24.22)	1472(23.53)	1519(24.28)		
College or above	698(5.17)	298(42.69)	172(24.64)	132(18.91)	96(13.75)		
Marital status							
Never	1038(7.69)	493(47.50)	253(24.37)	175(16.86)	117(11.27)	343.497	<0.001
Married	11849(87.77)	2743(23.15)	2960(24.98)	3040(25.66)	3106(26.21)		
Divorced or widowed	613(4.54)	138(22.51)	160(26.1)	161(26.26)	154(25.12)		
Income per year(yuan)							
0~	1984(14.7)	479(24.14)	510(25.71)	504(25.45)	490(24.7)	18.102	0.034
5000~	2412(17.87)	627(26)	651(26.99)	577(23.92)	557(23.09)		
10000~	3885(28.78)	969(24.94)	983(25.3)	962(24.76)	971(24.99)		
20000~	5219(38.66)	1299(24.89)	1229(23.55)	1332(25.52)	1359(26.04)		
Smoking status							
Current	3335(24.7)	698(20.93)	830(24.89)	824(24.71)	983(29.48)	110.976	<0.001
Former	821(6.08)	142(17.3)	201(24.48)	237(28.87)	241(29.35)		
Never	9344(69.21)	2534(27.12)	2342(25.06)	2315(24.78)	2153(23.04)		
Drinking status							
Current	3586(26.56)	663(18.49)	913(25.46)	931(25.96)	1079(30.09)	169.504	<0.001
Former	490(3.63)	88(17.96)	117(23.88)	129(26.33)	156(31.84)		
Never	9424(69.81)	2623(27.83)	2343(24.86)	2316(24.58)	2142(22.73)		
Body mass index, median(inter-quartile range)	22.66(4.33)	20.16(2.76)	21.93(3.05)	23.53(3.35)	25.53(3.69)	5508.028	<0.001

### 3.2 Risk of CVD according to MetS status and severity

As shown in **Table 2**, 541 participants were diagnosed with CVD (470 with stroke and 71 with heart disease) during the follow-up period (2010–2021). **Table 2** presents the incidence density of CVD among participants with different MetS status or scores and the association between MetS and incident CVD. The incidence density of CVD was 3.07 and 6.08 per 1000 person-years among participants without and with MetS, respectively. For the first (Q1), second (Q2), third (Q3), and fourth (Q4) quartiles of the MetS score, the incidence density of CVD was 1.62, 3.64, 3.65, and 5.90 per 1000 person-years, respectively(*P* for trend=0.037). After adjustment for age, gender, residence, educational level, marital status, annual income, BMI, smoking and drinking status, and the presence of MetS was related to a 1.7-fold higher risk of CVD (hazard ratio [HR]: 1.700, 95% confidence interval [CI]: 1.380–2.094), 1.56-fold higher risk of stroke (HR: 1.560, 95% CI: 1.245–1.954), and 2.83-fold higher risk of heart disease (HR: 2.828, 95% CI: 1.621–4.933).

**Table 2:** Incidence of cardiovascular disease according to metabolic syndrome(MetS) status.

Outcome	Cases,n	Incidence density, per 1000 person-years	HR(95%CI)		
			Model 1	Model 2	Model 3
Cardiovascular disease					
MetS status ^#^					
No	357	3.07	1[Reference]	1[Reference]	1[Reference]
Yes	184	6.08	1.998(1.676-2.381)	1.831(1.526-2.197)	1.700(1.380-2.094)
MetS score, quartile					
per unit increase	541	3.69	1.942(1.712-2.203)	1.729(1.514-1.975)	1.802(1.529-2.124)
Q1(<-0.432)	60	1.62	1[Reference]	1[Reference]	1[Reference]
Q2(-0.432~0.037)	133	3.64	2.253(1.661-3.056)	1.798(1.325-2.439)	1.812(1.329-2.470)
Q3(0.038~0.383)	134	3.65	2.258(1.665-3.062)	1.708(1.260-2.317)	1.746(1.265-2.410)
Q4(≥0.384)	214	5.90	3.654(2.744-4.866)	2.734(2.052-3.642)	2.817(2.015-3.938)
Stroke					
MetS status^#^					
No	317	2.73	1[Reference]	1[Reference]	1[Reference]
Yes	153	5.05	1.848(1.527-2.235)	1.662(1.364-2.026)	1.560(1.245-1.954)
MetS score, quartile					
per unit increase	470	3.21	1.844(1.608-2.115)	1.636(1.416-1.890)	1.726(1.443-2.064)
Q1(<-0.432)	53	1.43	1[Reference]	1[Reference]	1[Reference]
Q2(-0.432~0.037)	122	3.34	2.340(1.695-3.231)	1.864(1.350-2.574)	1.879(1.353-2.608)
Q3(0.038~0.383)	122	3.33	2.328(1.686-3.213)	1.758(1.274-2.429)	1.794(1.274-2.527)
Q4(≥0.384)	173	4.77	3.345(2.459-4.551)	2.496(1.834-3.397)	2.575(1.795-3.694)
Heart disease					
MetS status^#^					
No	40	0.34	1[Reference]	1[Reference]	1[Reference]
Yes	31	1.02	3.253(2.042-5.182)	3.317(2.053-5.360)	2.828(1.621-4.933)
MetS score, quartile					
per unit increase	71	0.48	2.652(1.930-3.645)	2.389(1.706-3.345)	2.260(1.498-3.410)
Q1(<-0.432)	7	0.19	1[Reference]	1[Reference]	1[Reference]
Q2(-0.432~0.037)	11	0.30	1.595(0.619-4.116)	1.274(0.494-3.288)	1.283(0.492-3.342)
Q3(0.038~0.383)	12	0.33	1.731(0.682-4.397)	1.298(0.510-3.300)	1.344(0.510-3.544)
Q4(≥0.384)	41	1.13	5.986(2.686-13.343)	4.505(2.017-10.060)	4.541(1.811-11.383)

Model 1 was crude model.

Model 2 was adjusted for age, gender, residence, education level, marital status and income.

Model 3 was adjusted as model 2 plus smoking status, drinking status and body mass index.

^#^Defined by the ATP-III criteria modified by American Diabetes Association (ADA).

**Figure 2:**
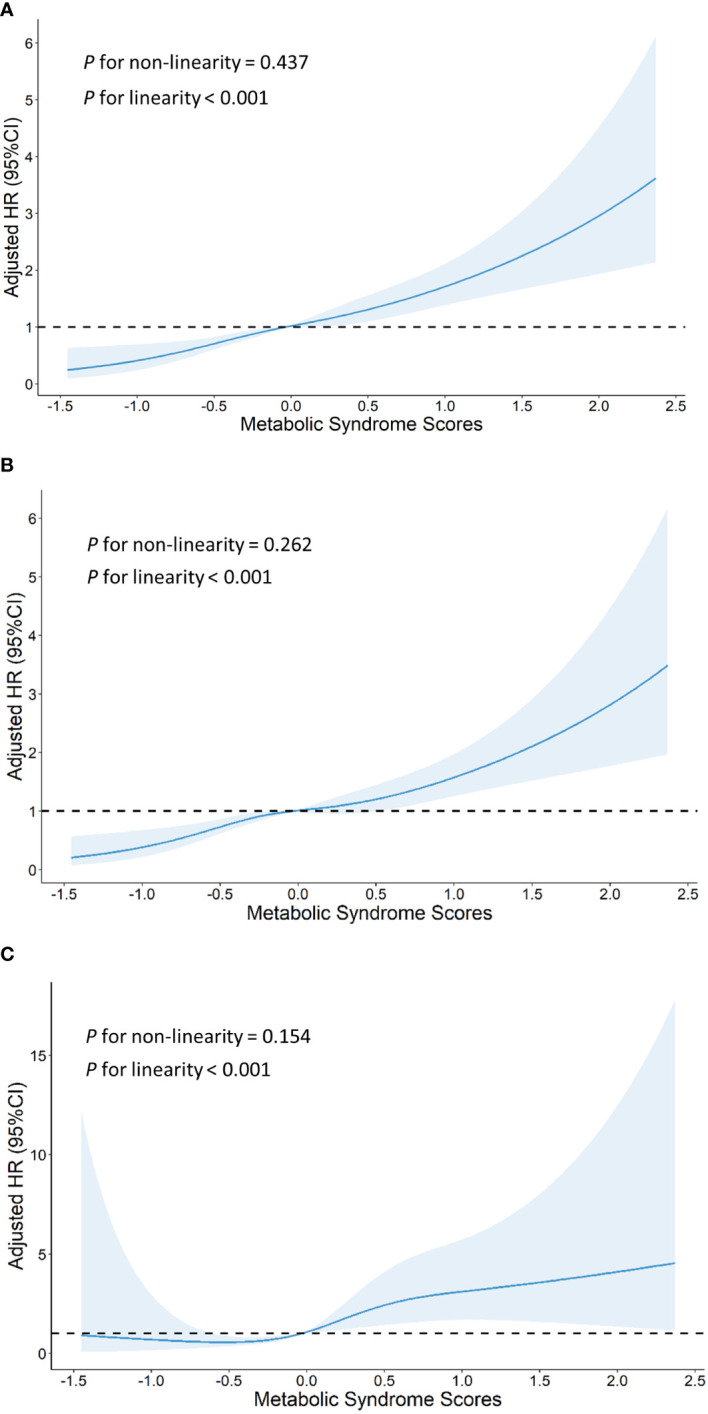
Graphs show HRs for cardiovascular disease **(A)**, stroke **(B)** and heart disease **(C)** adjusted for age, sex, residence, marital status, educational level, smoking status,drinking status and body mass index. Data were fitted by a restricted cubic spline Cox proportional hazards regression model. Solid lines indicate HRs, and shaded areas indicate 95% CIs.

Using the MetS score as continuous variable in the model, we found each unit increase in the MetS score was related to an 1.80-fold higher risk of CVD (HR: 1.802, 95% CI: 1.529–2.124), 1.73-fold higher risk of stroke(HR: 1.726, 95% CI: 1.443–2.064) and 2.26-fold higher risk of heart disease(HR: 2.260, 95% CI: 1.498–3.410). Compared with the HR (95% CI) for CVD events in the lowest quartile (Q1) of the MetS score, that in the Q2, Q3, and Q4 was 1.812 (1.329–2.470), 1.746 (1.265–2.410), and 2.817 (2.015–3.938), respectively (*P* for trend <0.001). Compared with the HR (95% CI) for stroke in the Q1 of the MetS score, that in the Q2, Q3, and Q4 was 1.879 (1.353–2.608), 1.794 (1.274–2.527), and 2.575 (1.795–3.694), respectively (*P* for trend <0.001). Compared to participants in the lowest quartiles of the MetS score, those in the highest quartiles of MetS score had a 4.54-fold higher risk of heart disease (HR: 4.541, 95% CI: 1.811–11.383) (*P* for trend <0.001). By using restricted cubic spline regression, a linear and positive correlation between the MetS score and CVD risk was observed in **Figure 2**, indicating a dose-response relationship. (for non-linearity, *P* = 0.437, 0.262, and 0.154 for CVD, stroke, and heart disease, respectively). In addition, the results of individual MetS component’s contribution to CVD were showed in **Table 3**. After the partial chi-square statistic minus the predicted degrees of freedom, MAP was the most critical factor for CVD(*x* ^2^-df=82.621) and stroke(*x* ^2^-df=83.216), followed by FBG(*x* ^2^-df=35.729 and 29.427 for CVD and stroke, respectively).FBG was the most critical factor for heart disease(*x* ^2^-df=4.430).

**Table 3:** The contribution of individual component of metabolic syndrome to cardiovascular disease.

Items	cardiovascular disease		stroke		heart disease	
	HR (95%CI)	*x* ^2^-df	HR (95%CI)	*x* ^2^-df	HR (95%CI)	*x* ^2^-df
WC	1.006(0.989-1.022)	-0.544	1.001(0.984-1.019)	-0.977	1.032(0.988-1.078)	1.038
MAP	1.033(1.026-1.040)	82.621	1.035(1.028-1.043)	83.216	1.018(0.997-1.039)	1.876
TC	0.934(0.859-1.015)	1.613	0.914(0.832-1.005)	2.450	1.024(0.866-1.211)	-0.922
HDL-C	0.900(0.634-1.279)	-0.656	0.935(0.643-1.359)	-0.875	0.645(0.232-1.792)	-0.294
FBG	1.151(1.100-1.205)	35.729	1.149(1.094-1.208)	29.427	1.152(1.023-1.297)	4.430

Model was adjusted for the five components of MetS, age, sex, residence, education level, marital status, income, smoking status, drinking status and body mass index.

WC, Waist circumference; MAP, Mean arterial pressure; TC, Triglycerides; HDL-C, High-density lipoprotein-cholesterol; FBG, Fasting blood glucose; HR, hazard ratio.

### 3.3 Subgroup and sensitivity analyses

**Figure 3:**
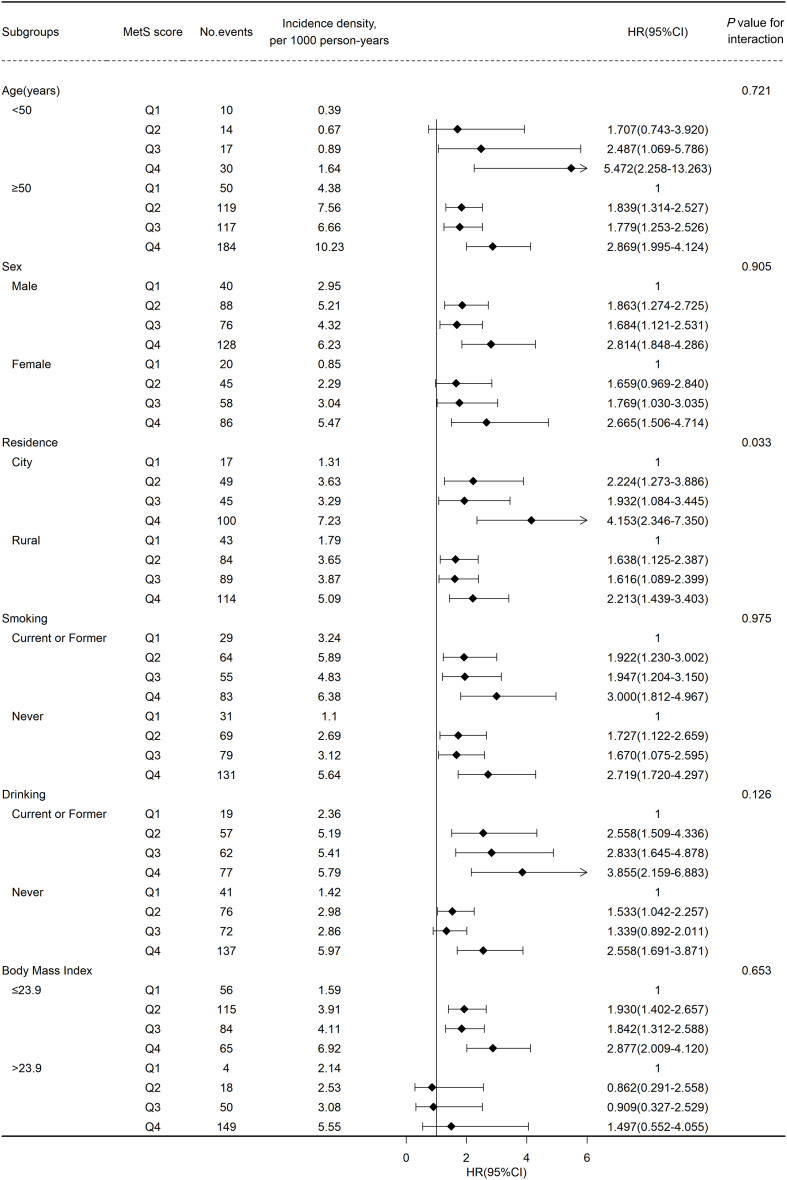
Association between metabolic syndrome(MetS) score and cardiovascular disease events risk stratified by different factors.

**Figure 3** shows the results of subgroup analyses. Generally, the positive correlation between MetS severity score and the risk of CVD remained robust after stratified by baseline characteristics. No significant interaction was found across age(<50 or ≥50), gender, BMI(≤22 or >22), smoking and drinking status. Notably, the correlation between MetS severity score and the risk of CVD was stronger on participants living in the city (Q1, reference; HR [95% CI] of Q2, 2.224 [1.273–3.886]; Q3, 1.932 [1.084–3.445]; Q4, 4.153 [2.346–7.350]) than on those living in rural areas (Q1, reference; HR [95% CI] of Q2, 1.638 [1.125–2.387]; Q3, 1.616 [1.089–2.399]; Q4, 2.213 [1.439–3.403]) at baseline (*P* for interaction = 0.033). In addition, similar results were found in the sensitivity analyses using a complete dataset, or by excluding events that occurred within the first 2 years of follow-up, or using competing risk models, suggesting a higher MetS score contributed to a higher risk of CVD, including stroke and heart disease (**Supplementary Material**: **Supplementary Tables S2-S4**).

## 4 Discussion

The present study computed the MetS severity score to quantify MetS severity and explored the association between MetS severity and the risk of CVD in a cohort of 13,500 Chinese adults with over 10 years of follow-up. We found that participants with MetS experienced an increased risk of incident CVD compared to those without MetS. Furthermore, a linear relationship was observed between the MetS severity score and CVD risk, indicating that a higher MetS severity score contributed to a higher risk of CVD. In addition, the positive correlation between the MetS severity and CVD risk was more pronounced in urban populations.

The correlation between MetS and CVD is supported by an increasing number of epidemiological studies. A cohort study conducted in Iran reported that MetS was related to an increased risk of coronary heart disease(HR:1.8; 95%CI:1.42–2.28) (27). A meta-analysis including 116,496 participants suggested that MetS increases the risk of incident stroke by 70% (11). The Rotterdam Study also found that patients with MetS were more likely to develop coronary heart disease, stroke, and cardiovascular mortality (28). A recent cohort study with a follow-up of 13 years reported that MetS was related to an 1.3-fold increase in CVD risk (29). As expected, in the present study, the presence of MetS was significantly associated with the risk of CVD (HR, 1.700; 95% CI: 1.380–2.094), including stroke and heart disease, among Chinese adults.

In recent decades, many studies have regarded MetS as a binomial variable, regardless of its severity. In recent years, accumulating studies have advocated evaluating MetS severity and have attempted to develop a continuous MetS scoring system. Notably, several MetS scoring systems have been derived from traditional MetS components through PC or confirmatory factor analysis (15–17, 30). In the present study, considering the different weights of MetS components, we applied a previously described continuous MetS severity scoring system widely used in epidemiological studies (9, 15, 31, 32). However, to the best of our knowledge, only a few studies have focused on the relationship between MetS severity and CVD risk. Nima et al. found a positive correlation between the MetS severity and composite CVD events, whereas the traditional definition of MetS did not indicate any significant association (33). In the Communities Study and Jackson Heart Study, the MetS severity score supplemented the ability to predict the risk of coronary heart disease (34). A nationwide population-based study in Korea also reported that a higher MetS severity Z-score was related to a higher risk of both myocardial infarction and stroke (20). The Kailuan cohort study, a relevant study conducted in China, reported a two-fold increased risk of CVD when comparing the 75^th^ and 25^th^ percentiles of the MetS severity score and observed a positive linear relationship, consistent with the results of the present study (18). In our study, the results of treating MetS as a continuous variable was aligned to dividing MetS scores into four categories in the multivariate-adjusted model, revealing that the higher the MetS score, the higher the risk of CVD. These results indicate that a quantitative assessment of MetS severity, rather than a simple dichotomous classification, may be more useful for accurately predicting CVD risk in the future. The continuous MetS score also makes it feasible to monitor MetS severity dynamically and suggests that participants can be classified according to MetS severity and different interventions can be implemented to prevent CVD.

The underlying mechanisms of the association between MetS and CVD remain unclear; however, some potential pathophysiological mechanisms have been suggested. For example, chronic overweight and obesity can cause the overactivation of the sympathetic nervous system, which concurrently leads to abnormalities in vascular function, cardiac function, and metabolic balance (35, 36). Hypertension may promote the process of left ventricular remodeling, including enlargement of left ventricular volume, thickening of the left ventricular wall and dilation of the left atrium. These changes will increase myocardial oxygen consumption and reduce diastolic perfusion time and volume, resulting in myocardial anoxia (37). Previous studies have found that increased MetS severity is associated with accelerated atherosclerosis, leading to an elevated risk of myocardial infarction (12). In addition, MetS can change the structure of microvascular and lead to microvascular dysfunction. And in this situation, the brain and heart may experience hypoxia and metabolic abnormalities due to insufficient blood supply, which furthermore increases the risk of CVD (38, 39). The findings of this study support the causal relationship between MetS and CVD from an epidemiological perspective, Notably, we found the positive correlation between the MetS severity score and CVD risk was more pronounced in urban populations, possibly due to urban residents having lower levels of physical activity or being more likely to be over-nourished or exposed to more pollution than rural residents (40–42).

This study adopted prospective design with a follow-up period of over 10 years, thus causal inferences was feasible. Unlike the Kailuan Cohort study, which mainly included employees from the Kailuan Company (43), the present study is the first cohort study to focus on the contribution of MetS severity to incident CVD in a provincially representative community-based population in China. However, this study also has some limitations. First, though we adjusted for a series of confounding factors, some potential factors that may affect the association between MetS and CVD, such as dietary patterns, physical activity, occupation, and air pollution (44, 45), were not considered. Second, owing to the limited sample size, we did not collect enough data on heart disease events; therefore, the estimation of the effects of the MetS severity score on heart disease was not sufficiently accurate. Third, the characteristics and lifestyles of participants may change over time; however, we only collected baseline information in the present study, which may have affected the results. Finally, the present study involved only Chinese participants; therefore, the findings may not be generalizable to other countries or ethnicities.

## 5 Conclusions

The presence of MetS was significantly related to an increased risk of CVD and its subtypes (stroke and heart disease), and a positive linear association was observed between MetS severity and CVD risk. These results indicate that assessing MetS severity and further ensuring intervention measures according to the different severity of MetS may be more useful for preventing and managing CVD.

## Data availability statement

The raw data supporting the conclusions of this article will be made available by the authors, without undue reservation.

## Ethics statement

The studies involving humans were approved by Ethics Committee of Zhejiang Provincial Center for Disease Control and Prevention. The studies were conducted in accordance with the local legislation and institutional requirements. The participants provided their written informed consent to participate in this study.

## Author contributions

J-JL: Writing – original draft, Methodology. P-YD: Data curation, Writing – review & editing. JZ: Data curation, Writing – review & editing. Y-QG: Data curation, Writing – review & editing. W-WG: Data curation, Investigation, Writing – review & editing. MY: Project administration, Writing – review & editing. LF: Investigation, Writing – review & editing. R-YH: Supervision, Writing – review & editing. Q-FH: Data curation, Writing – review & editing. NL: Data curation, Writing – review & editing. L-XW: Investigation, Writing – review & editing. M-BL: Investigation, Writing – review & editing. J-MZ: Funding acquisition, Supervision, Writing – review & editing.

## References

[1] Grundy SM Cleeman JI Daniels SR Donato KA Eckel RH Franklin BA. Diagnosis and management of the metabolic syndrome: an American Heart Association/National Heart, Lung, and Blood Institute Scientific Statement. Circulation. (2005) 112:2735–52. doi: 10.1161/CIRCULATIONAHA.105.169404 16157765

[2] Fahed G Aoun L Bou ZM Allam S Bou ZM Bouferraa Y. Metabolic syndrome: updates on pathophysiology and management in 2021. Int J Mol Sci. (2022) 23:786. doi: 10.3390/ijms23020786 35054972 PMC8775991

[3] Ranasinghe P Mathangasinghe Y Jayawardena R Hills AP Misra A. Prevalence and trends of metabolic syndrome among adults in the asia-pacific region: a systematic review. BMC Public Health. (2017) 17:101. doi: 10.1186/s12889-017-4041-1 28109251 PMC5251315

[4] Ansarimoghaddam A Adineh HA Zareban I Iranpour S HosseinZadeh A Kh F. Prevalence of metabolic syndrome in Middle-East countries: Meta-analysis of cross-sectional studies. Diabetes Metab Syndr. (2018) 12:195–201. doi: 10.1016/j.dsx.2017.11.004 29203060

[5] Hirode G Wong RJ. Trends in the prevalence of metabolic syndrome in the United States, 2011-2016. Jama. (2020) 323:2526–28. doi: 10.1001/jama.2020.4501 PMC7312413 32573660

[6] Yao F Bo Y Zhao L Li Y Ju L Fang H. Prevalence and influencing factors of metabolic syndrome among adults in China from 2015 to 2017. Nutrients. (2021) 13:4475. doi: 10.3390/nu13124475 34960027 PMC8705649

[7] Low S Khoo K Wang J Irwan B Sum CF Subramaniam T. Development of a metabolic syndrome severity score and its association with incident diabetes in an Asian population-results from a longitudinal cohort in Singapore. Endocrine. (2019) 65:73–80. doi: 10.1007/s12020-019-01970-5 31161560

[8] Kim J Park EY Park E Lim MK Oh JK Kim B. Metabolic syndrome and colorectal cancer risk: results of propensity score-based analyses in a community-based cohort study. Int J Environ Res Public Health. (2020) 17:8687. doi: 10.3390/ijerph17228687 33238496 PMC7700241

[9] Wu M Shu Y Wang L Song L Chen S Liu Y. Metabolic syndrome severity score and the progression of CKD. Eur J Clin Invest. (2022) 52:e13646. doi: 10.1111/eci.13646 34197633

[10] Tune JD Goodwill AG Sassoon DJ Mather KJ. Cardiovascular consequences of metabolic syndrome. Transl Res. (2017) 183:57–70. doi: 10.1016/j.trsl.2017.01.001 28130064 PMC5393930

[11] Li X Li X Lin H Fu X Lin W Li M. Metabolic syndrome and stroke: A meta-analysis of prospective cohort studies. J Clin Neurosci. (2017) 40:34–8. doi: 10.1016/j.jocn.2017.01.018 28268148

[12] Mottillo S Filion KB Genest J Joseph L Pilote L Poirier P. The metabolic syndrome and cardiovascular risk a systematic review and meta-analysis. J Am Coll Cardiol. (2010) 56:1113–32. doi: 10.1016/j.jacc.2010.05.034 20863953

[13] DeBoer MD Gurka MJ. Clinical utility of metabolic syndrome severity scores: considerations for practitioners. Diabetes Metab Syndr Obes. (2017) 10:65–72. doi: 10.2147/DMSO.S101624 28255250 PMC5325095

[14] Ragland DR. Dichotomizing continuous outcome variables: dependence of the magnitude of association and statistical power on the cutpoint. Epidemiology. (1992) 3:434–40. doi: 10.1097/00001648-199209000-00009 1391136

[15] Wijndaele K Beunen G Duvigneaud N Matton L Duquet W Thomis M. A continuous metabolic syndrome risk score: utility for epidemiological analyses. Diabetes Care. (2006) 29:2329. doi: 10.2337/dc06-1341 17003322

[16] Wiley JF Carrington MJ. A metabolic syndrome severity score: a tool to quantify cardio-metabolic risk factors. Prev Med. (2016) 88:189–95. doi: 10.1016/j.ypmed.2016.04.006 27095322

[17] Graziano F Grassi M Sacco S Concas MP Vaccargiu S Pirastu M. Probing the factor structure of metabolic syndrome in Sardinian genetic isolates. Nutr Metab Cardiovasc Dis. (2015) 25:548–55. doi: 10.1016/j.numecd.2015.02.004 25836955

[18] Tang X Wu M Wu S Tian Y. Continuous metabolic syndrome severity score and the risk of CVD and all-cause mortality. Eur J Clin Invest. (2022) 52:e13817. doi: 10.1111/eci.13817 35598176

[19] Lee EY Han K Kim DH Park YM Kwon HS Yoon KH. Exposure-weighted scoring for metabolic syndrome and the risk of myocardial infarction and stroke: a nationwide population-based study. Cardiovasc Diabetol. (2020) 19:153. doi: 10.1186/s12933-020-01129-x 32993664 PMC7525999

[20] Jang YN Lee JH Moon JS Kang DR Park SY Cho J. Metabolic syndrome severity score for predicting cardiovascular events: a nationwide population-based study from Korea. Diabetes Metab J. (2021) 45:569–77. doi: 10.4093/dmj.2020.0103 PMC8369214 33516167

[21] DeBoer MD Filipp SL Sims M Musani SK Gurka MJ. Risk of ischemic stroke increases over the spectrum of metabolic syndrome severity. Stroke. (2020) 51:2548–52. doi: 10.1161/STROKEAHA.120.028944 PMC7428063 32552367

[22] Gurka MJ Guo Y Filipp SL DeBoer MD. Metabolic syndrome severity is significantly associated with future coronary heart disease in Type 2 diabetes. Cardiovasc Diabetol. (2018) 17:17. doi: 10.1186/s12933-017-0647-y 29351794 PMC5775549

[23] Hao W Xinwei Z Jie Z Qingfang H Ruying H Lixin W. Factors associated with prevalence, awareness, treatment and control of hypertension among adults in southern China: a community-based, cross-sectional survey. PloS One. (2013) 8:e62469. doi: 10.1371/journal.pone.0062469 23671599 PMC3650037

[24] von Elm E Altman DG Egger M Pocock SJ Gotzsche PC Vandenbroucke JP. The strengthening the reporting of observational studies in epidemiology (STROBE) statement: guidelines for reporting observational studies. Int J Surg. (2014) 12:1495–99. doi: 10.1016/j.ijsu.2014.07.013

[25] Gong W Wei X Liang Y Zou G Hu R Deng S. Urban and rural differences of acute cardiovascular disease events: a study from the population-based real-time surveillance system in Zhejiang, China in 2012. PloS One. (2016) 11:e165647. doi: 10.1371/journal.pone.0165647 PMC5089742 27802321

[26] Fine JP GR. A proportional hazards model for the subdistribution of a competing risk. J Am Stat Assoc. (1999) 446:496–509. doi: 10.1080/01621459.1999.10474144

[27] Esteghamati A Hafezi-Nejad N Sheikhbahaei S Heidari B Zandieh A Ebadi M. Risk of coronary heart disease associated with metabolic syndrome and its individual components in Iranian subjects: a matched cohort study. J Clin Lipidol. (2014) 8:279–86. doi: 10.1016/j.jacl.2014.02.002 24793349

[28] van Herpt TT Dehghan A van Hoek M Ikram MA Hofman A Sijbrands EJ. The clinical value of metabolic syndrome and risks of cardiometabolic events and mortality in the elderly: the Rotterdam study. Cardiovasc Diabetol. (2016) 15:69. doi: 10.1186/s12933-016-0387-4 27117940 PMC4847340

[29] Guembe MJ Fernandez-Lazaro CI Sayon-Orea C Toledo E Moreno-Iribas C. Risk for cardiovascular disease associated with metabolic syndrome and its components: a 13-year prospective study in the RIVANA cohort. Cardiovasc Diabetol. (2020) 19:195. doi: 10.1186/s12933-020-01166-6 33222691 PMC7680587

[30] Gurka MJ Lilly CL Oliver MN DeBoer MD. An examination of sex and racial/ethnic differences in the metabolic syndrome among adults: a confirmatory factor analysis and a resulting continuous severity score. Metabolism. (2014) 63:218–25. doi: 10.1016/j.metabol.2013.10.006 PMC4071942 24290837

[31] Lourenco LP Viola P Franceschini S Rosa C Ribeiro S. Metabolic syndrome and risk factors in children: a risk score proposal. Eur J Clin Nutr. (2023) 77:278–82. doi: 10.1038/s41430-022-01217-z 36203009

[32] Madden KM Feldman B Chase J. Sedentary time and metabolic risk in extremely active older adults. Diabetes Care. (2021) 44:194–200. doi: 10.2337/dc20-0849 33067259 PMC7783925

[33] Motamed N Ajdarkosh H Karbalaie NM Panahi M Farahani B Rezaie N. Scoring systems of metabolic syndrome and prediction of cardiovascular events: A population based cohort study. Clin Cardiol. (2022) 45:641–49. doi: 10.1002/clc.23827 PMC9175260 35419856

[34] Guo Y Musani SK Sims M Pearson TA DeBoer MD Gurka MJ. Assessing the added predictive ability of a metabolic syndrome severity score in predicting incident cardiovascular disease and type 2 diabetes: the Atherosclerosis Risk in Communities Study and Jackson Heart Study. Diabetol Metab Syndr. (2018) 10:42. doi: 10.1186/s13098-018-0344-3 29796112 PMC5956946

[35] Ciccarelli M Santulli G Pascale V Trimarco B Iaccarino G. Adrenergic receptors and metabolism: role in development of cardiovascular disease. Front Physiol. (2013) 4:265. doi: 10.3389/fphys.2013.00265 24106479 PMC3789271

[36] Grassi G Seravalle G Quarti-Trevano F Scopelliti F Dell'Oro R Bolla G. Excessive sympathetic activation in heart failure with obesity and metabolic syndrome: characteristics and mechanisms. Hypertension. (2007) 49:535–41. doi: 10.1161/01.HYP.0000255983.32896.b9 17210829

[37] Unger T Borghi C Charchar F Khan NA Poulter NR Prabhakaran D. 2020 International society of hypertension global hypertension practice guidelines. Hypertension. (2020) 75:1334–57. doi: 10.1161/HYPERTENSIONAHA.120.15026 32370572

[38] Chantler PD Shrader CD Tabone LE D'Audiffret AC Huseynova K Brooks SD. Cerebral cortical microvascular rarefaction in metabolic syndrome is dependent on insulin resistance and loss of nitric oxide bioavailability. Microcirculation. (2015) 22:435–45. doi: 10.1111/micc.12209 PMC4551443 26014499

[39] Yang Z Liu Y Li Z Feng S Lin S Ge Z. Coronary microvascular dysfunction and cardiovascular disease: Pathogenesis, associations and treatment strategies. BioMed Pharmacother. (2023) 164:115011. doi: 10.1016/j.biopha.2023.115011 37321056

[40] Wang Y Wang Y Xu H Zhao Y Marshall JD. Ambient air pollution and socioeconomic status in China. Environ Health Perspect. (2022) 130:67001. doi: 10.1289/EHP9872 35674427 PMC9175641

[41] Zhu W Chi A Sun Y. Physical activity among older Chinese adults living in urban and rural areas: A review. J Sport Health Sci. (2016) 5:281–86. doi: 10.1016/j.jshs.2016.07.004 PMC6188614 30356525

[42] KunPLiuYPeiXLuoH. Regional and urban-rural disparities in prevalence of over-weight among old people in China: evidence from four Chinese provinces. J Nutr Health Aging. (2013) 17:859–64. doi: 10.1007/s12603-013-0343-x 24257569

[43] Wang L Lee Y Wu Y Zhang X Jin C Huang Z. A prospective study of waist circumference trajectories and incident cardiovascular disease in China: the Kailuan Cohort Study. Am J Clin Nutr. (2021) 113:338–47. doi: 10.1093/ajcn/nqaa331 33330917

[44] Liu F Wang X Pan M Zhang K Zhou F Tong J. Exposure to air pollution and prevalence of metabolic syndrome: A nationwide study in China from 2011 to 2015. Sci Total Environ. (2023) 855:158596. doi: 10.1016/j.scitotenv.2022.158596 36089046

[45] Gallardo-Alfaro L Bibiloni M Mascaro CM Montemayor S Ruiz-Canela M Salas-Salvado J. Leisure-time physical activity, sedentary behaviour and diet quality are associated with metabolic syndrome severity: The PREDIMED-Plus Study. Nutrients. (2020) 12:1013. doi: 10.3390/nu12041013 32272653 PMC7230557

