# Supplementary material for: Association between metabolic syndrome severity score and cardiovascular disease: results from a longitudinal cohort study on Chinese adults
Source: Front Endocrinol (Lausanne). 2024 Apr 8;15:1341546. doi: 10.3389/fendo.2024.1341546 (PMC11036864; doi:10.3389/fendo.2024.1341546)
Supplement: Supplementary file 1 [file Table_1.docx]

**Supplementary material**

Table S1. Baseline Characteristics Between Participants Included and excluded

| Characteristics | | Excluded | Included | *P* value |
| --- | --- | --- | --- | --- |
| Number of participants | | 645 | 12855 |  |
| Age,year,median(inter-quartile range) | | 49(23) | 47(21) | 0.006 |
| Men, No.(%) | | 308(47.75) | 6099(47.44) | 0.879 |
| City residence, No.(%) | | 500(77.52) | 4482(34.87) | <0.001 |
| Education level, No.(%) | |  |  | <0.001 |
|  | No formal education | 162(25.39) | 2092(16.27) |  |
|  | Primary school | 205(32.13) | 4084(31.77) |  |
|  | Secondary school | 236(36.99) | 6017(46.81) |  |
|  | College or above | 35(5.49) | 662(5.15) |  |
| Marrital status, No.(%) | |  |  | 0.107 |
|  | Never | 36(5.62) | 1002(7.79) |  |
|  | Married | 572(89.24) | 11273(87.69) |  |
|  | Divorced or widowed | 33(5.15) | 580(4.51) |  |
| Income per year（yuan), No.(%) | |  |  | 0.152 |
|  | 0~ | 8(28.57) | 1889(14.70) |  |
|  | 5000~ | 4(14.29) | 2310(17.97) |  |
|  | 10000~ | 9(32.14) | 3708(28.85) |  |
|  | 20000~ | 7(25.00) | 4947(38.49) |  |
| Smoking status, No.(%) | |  |  | 0.001 |
|  | Current | 122(18.91) | 3213(24.99) |  |
|  | Former | 34(5.27) | 787(6.12) |  |
|  | Never | 489(75.81) | 8855(68.88) |  |
| Drinking status, No.(%) | |  |  | 0.448 |
|  | Current | 174(26.98) | 3412(26.54) |  |
|  | Former | 29(4.50) | 461(3.59) |  |
|  | Never | 442(68.35) | 8982(69.87) |  |
| Body mass index, kg/m2,median(inter-quartile range) | | 22.99(4.60) | 22.64(4.31) | 0.038 |
| WC, cm, median(inter-quartile range) | | 80.85(13.67) | 79.45(12.95) | <0.001 |
| SBP, mmHg, median(inter-quartile range) | | 123(26.80) | 120(21) | <0.001 |
| DBP, mmHg, median(inter-quartile range) | | 80(13) | 78(12) | <0.001 |
| TC, mmol/L, median(inter-quartile range) | | 1.11(0.93) | 1.09(0.86) | <0.001 |
| HDL-C, mmol/L, median(inter-quartile range) | | 1.12(0.34) | 1.09(0.32) | 0.951 |
| FBG, mmol/L, median(inter-quartile range) | | 4.92(0.85) | 4.8(0.60) | <0.001 |

Abbreviations: WC,Waist circumference; SBP, systolic blood pressure; DBP, diastolic blood pressure; TC,Triglycerides; HDL-C,High-density lipoprotein-cholesterol; FBG,Fasting blood glucose

**Table S2. Association of** **Metabolic Syndrome(MetS) Status With Cardiovascular Diseases in Subpopulations of 12 855 Participants With Complete Data**

|  |  | HR(95%CI) |  |  |
| --- | --- | --- | --- | --- |
| Outcome | | Model 1 | Model 2 | Model 3 |
| Cardiovascular disease | |  |  |  |
| MetS status# | |  |  |  |
|  | No | 1[Reference] | 1[Reference] | 1[Reference] |
|  | Yes | 2.010(1.678-2.408) | 1.846(1.529-2.227） | 1.751(1.412-2.171) |
| MetS scores,quintile | |  |  |  |
|  | Q1(<-0.432) | 1[Reference] | 1[Reference] | 1[Reference] |
|  | Q2(-0.432~0.037) | 2.222(1.633-3.024) | 1.794(1.318-2.442) | 1.824(1.333-2.496) |
|  | Q3(0.038~0.383) | 2.210(1.624-3.009) | 1.690(1.241-2.302) | 1.754(1.265-2.433) |
|  | Q4(≥0.384) | 3.505(2.621-4.689) | 2.667(1.992-3.569) | 2.811(1.997-3.957) |
| Stroke | |  |  |  |
| MetS status | |  |  |  |
|  | No | 1[Reference] | 1[Reference] | 1[Reference] |
|  | Yes | 1.893(1.558-2.300) | 1.715(1.400-2.101) | 1.643(1.303-2.072) |
| MetS scores,quintile | |  |  |  |
|  | Q1(<-0.432) | 1[Reference] | 1[Reference] | 1[Reference] |
|  | Q2(-0.432~0.037) | 2.307(1.665-3.196) | 1.860(1.342-2.578) | 1.892(1.357-2.637) |
|  | Q3(0.038~0.383) | 2.293(1.654-3.179) | 1.752(1.263-2.430) | 1.816(1.283-2.570) |
|  | Q4(≥0.384) | 3.272(2.394-4.471) | 2.482(1.815-3.395) | 2.622(1.815-3.787) |
| Heart disease | |  |  |  |
| MetS status | |  |  |  |
|  | No | 1[Reference] | 1[Reference] | 1[Reference] |
|  | Yes | 3.000(1.834-4.907) | 3.003(1.808-4.988) | 2.617(1.453-4.714) |
| MetS scores,quintile | |  |  |  |
|  | Q1(<-0.432) | 1[Reference] | 1[Reference] | 1[Reference] |
|  | Q2(-0.432~0.037) | 1.596(0.619-4.116) | 1.296(0.502-3.344) | 1.308(0.501-3.415) |
|  | Q3(0.038~0.383) | 1.599(0.620-4.125) | 1.222(0.473-3.155) | 1.287(0.478-3.466) |
|  | Q4(≥0.384) | 5.238(2.327-11.792) | 4.043(1.792-9.124) | 4.168(1.623-10.702) |

Model 1 was crude model

Model 2 was adjusted for age, gender,residence,education level,marrital status and income.

Model 3 was adjusted as model 2 plus smoking status,drinking status and body mass index.

^#^Defined by the ATP-III criteria modified by American Diabetes Association(ADA)

**Table S3. Association of Metabolic Syndrome(MetS) Status With Cardiovascular Diseases in 13361 Participants excluding CVD events occured within the first two years of follow-up**

|  |  | HR(95%CI) |  |  |
| --- | --- | --- | --- | --- |
| Outcome | | Model 1 | Model 2 | Model 3 |
| Cardiovascular disease | |  |  |  |
| MetS status^#^ | |  |  |  |
|  | No | 1[Reference] | 1[Reference] | 1[Reference] |
|  | Yes | 1.956(1.625-2.355) | 1.788(1.475-2.168） | 1.631(1.309-2.032) |
| MetS scores,quintile | |  |  |  |
|  | Q1(<-0.432) | 1[Reference] | 1[Reference] | 1[Reference] |
|  | Q2(-0.432~0.037) | 2.226(1.639-3.134) | 1.807(1.307-2.499) | 1.823(1.312-2.534) |
|  | Q3(0.038~0.383) | 2.311(1.673-3.191) | 1.745(1.263-2.410) | 1.783(1.267-2.509) |
|  | Q4(≥0.384) | 3.778(2.789-5.119) | 2.818(2.079-3.820) | 2.907(2.039-4.146) |
| Stroke | |  |  |  |
| MetS status | |  |  |  |
|  | No | 1[Reference] | 1[Reference] | 1[Reference] |
|  | Yes | 1.819(1.489-2.222) | 1.640(1.332-2.019) | 1.496(1.181-1.895) |
| MetS scores,quintile | |  |  |  |
|  | Q1(<-0.432) | 1[Reference] | 1[Reference] | 1[Reference] |
|  | Q2(-0.432~0.037) | 2.339(1.661-3.295) | 1.862(1.322-2.623) | 1.872(1.321-2.651) |
|  | Q3(0.038~0.383) | 2.412(1.716-3.391) | 1.821(1.295-2.561) | 1.846(1.286-2.649) |
|  | Q4(≥0.384) | 3.518(2.542-4.869) | 2.617(1.890-3.624) | 2.670(1.825-3.906) |
| Heart disease | |  |  |  |
| MetS status | |  |  |  |
|  | No | 1[Reference] | 1[Reference] | 1[Reference] |
|  | Yes | 3.205(1.923-5.343) | 3.203(1.890-5.427) | 2.871(1.555-5.301) |
| MetS scores,quintile | |  |  |  |
|  | Q1(<-0.432) | 1[Reference] | 1[Reference] | 1[Reference] |
|  | Q2(-0.432~0.037) | 1.696(0.616-4.667) | 1.357(0.493-3.735) | 1.404(0.505-3.904) |
|  | Q3(0.038~0.383) | 1.517(0.540-4.263) | 1.116(0.396-3.140) | 1.207(0.412-3.534) |
|  | Q4(≥0.384) | 5.814(2.441-13.847) | 4.374(1.833-10.438) | 4.803(1.769-13.044) |

Model 1 was crude model

Model 2 was adjusted for age, gender,residence,education level,marrital status and income.

Model 3 was adjusted as model 2 plus smoking status,drinking status and body mass index.

^#^Defined by the ATP-III criteria modified by American Diabetes Association(ADA)

**Table S4. Association of Metabolic Syndrome(MetS) Status With Cardiovascular Diseases in 13500 Participants using competing risk modle**

|  |  | HR(95%CI) |  |  |
| --- | --- | --- | --- | --- |
| Outcome | | Model 1 | Model 2 | Model 3 |
| Cardiovascular disease | |  |  |  |
| MetS status^#^ | |  |  |  |
|  | No | 1[Reference] | 1[Reference] | 1[Reference] |
|  | Yes | 2.000(1.680-2.390) | 1.874(1.559-2.253） | 1.717(1.385-2.128) |
| MetS scores,quintile | |  |  |  |
|  | Q1(<-0.432) | 1[Reference] | 1[Reference] | 1[Reference] |
|  | Q2(-0.432~0.037) | 2.240(1.650-3.040) | 1.818(1.339-2.469) | 1.820(1.332-2.488) |
|  | Q3(0.038~0.383) | 2.260(1.660-3.060) | 1.756(1.292-2.386) | 1.779(1.283-2.466) |
|  | Q4(≥0.384) | 3.650(2.740-4.860) | 2.813(2.109-3.753) | 2.845(2.026-3.995) |
| Stroke | |  |  |  |
| MetS status | |  |  |  |
|  | No | 1[Reference] | 1[Reference] | 1[Reference] |
|  | Yes | 1.850(1.530-2.240) | 1.704(1.397-2.079) | 1.579(1.251-1.992) |
| MetS scores,quintile | |  |  |  |
|  | Q1(<-0.432) | 1[Reference] | 1[Reference] | 1[Reference] |
|  | Q2(-0.432~0.037) | 2.330(1.690-3.210) | 1.883(1.362-2.603) | 1.889(1.356-2.629) |
|  | Q3(0.038~0.383) | 2.330(1.690-3.210) | 1.806(1.306-2.498) | 1.831(1.295-2.590) |
|  | Q4(≥0.384) | 3.340(2.460-4.540) | 2.572(1.888-3.504) | 2.611(1.804-3.779) |
| Heart disease | |  |  |  |
| MetS status | |  |  |  |
|  | No | 1[Reference] | 1[Reference] | 1[Reference] |
|  | Yes | 3.260(2.050-5.190) | 3.379(2.082-5.485) | 2.824(1.635-4.875) |
| MetS scores,quintile | |  |  |  |
|  | Q1(<-0.432) | 1[Reference] | 1[Reference] | 1[Reference] |
|  | Q2(-0.432~0.037) | 1.590(0.615-4.100) | 1.298(0.504-3.342) | 1.283(0.491-3.350) |
|  | Q3(0.038~0.383) | 1.730(0.681-4.400) | 1.348(0.532-3.415) | 1.365(0.514-3.624) |
|  | Q4(≥0.384) | 5.980(2.682-13.300) | 4.614(2.066-10.307) | 4.512(1.904-10.690) |

Model 1 was crude model

Model 2 was adjusted for age, gender,residence,education level,marrital status and income.

Model 3 was adjusted as model 2 plus smoking status,drinking status and body mass index.

^#^ Defined by the ATP-III criteria modified by American Diabetes Association(ADA)
